# Changes in structuredness of cytoplasmic matrix in single stimulated lymphocytes from healthy donors and patients with non-malignant and malignant diseases.

**DOI:** 10.1038/bjc.1979.152

**Published:** 1979-07

**Authors:** Y. Hashimoto, F. Takaku, T. Yamanaka


Br. J. Cancer (1979) **40**, 156

***Short Communication***

### CHANGES IN STRUCTUREDNESS OF CYTOPLASMIC MATRIX IN SINGLE STIMULATED LYMPHOCYTES FROM HEALTHY DONORS AND PATIENTS WITH NON-MALIGNANT AND MALIGNANT DISEASES

Y. HASHIMOTO*, F. TAKAKU† AND T. YAMANAKA‡

*From *The Third Department of Internal Medicine, Faculty of Medicine, University of Tokyo, and †The First Department of Internal Medicine and ‡Department of Gastroenterology, Jichi Medical School, Tochigi, Japan*

Received 27 April 1978 Accepted 12 March 1979

CERCEK *et al.* (1974*b*) reported the changes in fluorescence polarization in cytoplasm of lymphocytes stimulated by various kinds of antigens. Lymphocytes from patients with malignant diseases were differentiated from those of healthy donors or donors with non-malignant diseases on the basis of changes in the structuredness of cytoplasmic matrix (SCM) induced by cancer basic protein (CaBP) and phytohaemoagglutinin (PHA) (Cercek *et al.*, 1974a). These observations were confirmed by us in patients with gastric cancer in early and in advanced stages (Takaku *et al.*, 1977). In those studies, the changes in SCM had been measured on cell suspensions, using a fluorescence spectrophotometer equipped with polarization accessories and a thermostatic cuvette holder. The data obtained by this method were, therefore, the average changes in SCM of whole cell populations.

Recently, Cercek & Cercek (1976) showed that the changes in SCM of single lymphocytes could be measured with a single-cell polarization meter composed of a fluorescence microscope, polarizing accessories and photomultipliers. The work described was intended to confirm Cercek's results and to improve the conditions for the assay of cancer antigens.

*Single-cell polarization meter*

We constructed a single-cell polarization meter with a Nikon SPM-RFL-P fluorescence microscope equipped with an epi-fluorescence condenser, and a Wallaston prism fitted between the 2 photomultipliers and the tube body. A 200W ultra-high-pressure mercury arc with a Glan-Thompson prism between the arc and a dichroic mirror was used in this system instead of a Xenon light. To prevent photobleaching of fluorescein molecules, an interference filter transmitting the light only between 460 nm and 480 nm was installed between the mercury arc and the excitation polarizer. A Glan-Thompson prism transmitting only vertically polarized light was also fitted. The epicondenser was attached with a barrier filter to cut off the light below 505 nm. In every measurement, a Nikon F1/40/1·30 glyc objective was used. The diameter of the measuring diagram in front of the Wallaston prism was adjusted to match the size of the lymphocyte image. The intensities of the emissions parallel, $I_{\parallel}$, and perpendicular, $I_{\perp}$, to the polarised exciting light were recorded on a 2-pen recorder. The fluorescence-polarization values (P values) were calculated from the relationship; $P = (I_{\parallel} - Q \cdot I_{\perp})/(I_{\parallel} + Q \cdot L_{\perp})$, where Q denotes a

Correspondence to: Yasuo Hashimoto, M.D., The Third Department of Internal Medicine, Faculty of Medicine, University of Tokyo, 7-3-1, Hongo, Bunkyo, Tokyo, Japan 113.

FIG. 1.—The relationship between the $P$ values and the concentration of glycerin in water–glycerin mixtures in the Hitachi MPF-4 fluorescence spectrophotometer (solid line) and the Nikon SPM-RFL-P single-cell polarization meter (dotted line).

FIG. 2.—The single-cell polarization meter. L: 200 W ultra-high-pressure mercury light, SH: shutter, $F_1$: interference filter transmitting light between 460 nm and 480 nm, P: Glan-Thompson prism, M: dichroic mirror, O: microscope objective, S: sample, $F_2$: cut filter, transmitting light above 505 nm, PH: changeable pinhole, WP: Wallaston prism, PM: photomultiplier.

correction factor for the unequal transmission of the 2 components of polarized light through the optical system. We adjusted the gain of each amplifier to obtain 1·0 for a correction factor, Q. The accuracy and ability of the optical system was checked by measuring the P value of water–glycerol mixtures of varying viscosities containing 5$\mu$M fluorescein at 25°C and comparing them with the values obtained by using the Hitachi MPF-4 fluorescence spectrophotometer. The relationship between P values and glycerol concentrations of water–glycerol mixtures was almost identical between these 2 systems, as shown in Fig. 1. The diagram of the optical system is presented in Fig. 2.

*Lymphocyte preparation*

Human lymphocytes were obtained from heparinized peripheral blood by the Ficoll-Triosil gradient separation method (Harris and Ukaejiofo, 1969; Cercek & Cercek, 1977). The density of Ficoll–Triosil gradients was 1·081 g/ml at 25°C. The lymphocytes were suspended in Dulbecco's phosphate-buffered saline at a concentration of $6 \times 10^6$ cells/ml.

*Lymphocyte stimulation*

Aliquots of 50 $\mu$l of the lymphocyte suspension were incubated for 60 min at 37°C with either 5 $\mu$l of 40× diluted PHA (Wellcome Ltd) or 5 $\mu$l of partially purified CaBP solution. The concentration of CaBP was 50 $\mu$g/ml. CaBP was purified from the tissue of rectal cancer obtained from a patient according to the method of Carnegie *et al.* (1973).

*Measurement of P values*

Aqueous solutions of fluorescein diacetate (FDA) in complete isotonic phosphate-buffered saline (pH 7·4, 280–290 mOsm) (Paul, 1970) were prepared by sequential dilutions of a stock solution of 25 mg of recrystallized FDA per ml of reagent-grade acetone. The final concentration of FDA used in the SCM measurement was 1·1 μM. Finally, acetone was diluted $5 \times 10^4$ times. The P values of fluorescein molecules (produced by enzymatic hydrolysis of the non-fluorescein substrate) in the cytoplasm of living cells were measured with the single-cell polarization meter. Ten μl of control or incubated lymphocyte suspensions ($\sim 6 \times 10^4$ lymphocytes) were mixed with 10 μl of 0·50μM FDA solution in complete PBS solution on a pre-cleaned microslide (Matsunami Glass Ind., Ltd). The samples were covered with pre-cleaned coverslip No. 1 (Matsunami Glass Ind., Ltd). The microslide and coverslip were confirmed as non-fluorescent. The recorder was started to record the values of $I_{\parallel}$ and $I_{\perp}$ when the sample was prepared. The measurement was carried out at 22°C. We did not measure under thermostatic conditions, but the room temperature was carefully controlled during the measurement. P values of about 200 single lymphocytes chosen at random were measured. Background fluoresence in this system was negligible when the diluted FDA solution was used.

The P values of individual unstimulated lymphocytes from 5 healthy donors and 5 patients each with non-malignant diseases and cancer ranged from 0·05 to 0·25, and the mean P values of each patient were from 0·13 to 0·19 (Table I). The mean P values of lymphocytes from these 3 kinds of donor before and after stimulation with PHA and CaBP are shown in Table I. All the P values were lower than those reported by Cercek & Cercek (1976), probably owing to the difference of conditions such as the time of measurement, the FDA concentrations, the osmolarity of Dulbecco's phosphate-buffered saline, and the characteristics of the optical system. As shown in Table I, almost all lymphocyte samples from healthy donors and patients with non-malignant diseases responded to PHA with decrease of P values but not to CaBP, whereas those from patients with cancer responded to CaBP, but not to PHA. As shown in Table II, the mean P values of individual lymphocytes were stable during the 10–30 min from the

TABLE I.—*Mean SCM values of lymphocytes from healthy donors and patients with malignant and non-malignant diseases*

| No. | Age | Sex | Diagnosis | $P_{CONTROL}$ | $P_{PHA}$ | $P_{CaBP}$ | $RR_{SCM}$ |
|---|---|---|---|---|---|---|---|
| 1 | 36 | male | Healthy | 0·1575 | 0·1212 | 0·1569 | 1·29 |
| 2 | 62 | male | Healthy | 0·1675 | 0·1289 | 0·1650 | 1·28 |
| 3 | 52 | male | Healthy | 0·1572 | 0·1321 | 0·1550 | 1·17 |
| 4 | 32 | female | Healthy | 0·1479 | 0·1079 | 0·1460 | 1·34 |
| 5 | 48 | male | Healthy | 0·1480 | 0·1201 | 0·1490 | 1·24 |
| 6 | 72 | male | Apoplexy | 0·1581 | 0·1321 | 0·1550 | 1·17 |
| 7 | 63 | male | Hypertension | 0·1520 | 0·1210 | 0·1530 | 1·26 |
| 8 | 45 | male | Liver cirrhosis | 0·1711 | 0·1424 | 0·1793 | 1·26 |
| | | | Acanthosis nigricans | | | | |
| 9 | 44 | female | Interstitial pneumonitis | 0·1394 | 0·1185 | 0·1370 | 1·16 |
| | | | Duodenal ulcer | | | | |
| 10 | 50 | female | Sjogren's syndrome | 0·1491 | 0·1327 | 0·1360 | 1·02 |
| | | | Scleroderma | | | | |
| 11 | 54 | male | Oesophageal Ca | 0·1306 | 0·1255 | 0·1206 | 0·96 |
| 12 | 41 | male | Stomach Ca | 0·1750 | 0·1707 | 0·1233 | 0·72 |
| 13 | 52 | male | Colon Ca | 0·1987 | 0·1862 | 0·1258 | 0·67 |
| 14 | 52 | male | Stomach Ca | 0·1536 | 0·1422 | 0·1153 | 0·81 |
| 15 | 55 | male | Kidney Ca | 0·1454 | 0·1408 | 0·1357 | 0·98 |

The blood samples were tested "blind", so to a limited extent this study confirms that SCM measurements can be used as a diagnostic test for cancer. However, it should be noted that the erythrocyte sedimentation rate of cancer patients is usually considerably higher than that of healthy subjects, so in principle the operator might have been able to distinguish between blood samples on this basis alone, in which case the test is not, strictly speaking, a completely blind trial. A more rigorous test would be to compare the results in cases of malignant and appropriate non-malignant pathology, with similar erythrocyte sedimentation rates.

TABLE II.—*Mean values of SCM in single lymphocytes after exposure to FDA*

| Time (min) | Polarization values ±s.d. |
|---|---|
| 0·5–5 | 0·2032±0·0462 |
| 5–10 | 0·1659±0·0292 |
| 10–15 | 0·1507±0·0186 |
| 15–20 | 0·1523±0·0126 |
| 20–25 | 0·1495±0·0242 |
| 25–30 | 0·1509±0·0090 |

The measurement of the polarization values of individual lymphocytes was started immediately after the lymphocyte suspension was mixed with Dulbecco's PBS containing 1μM FDA.

FIGURE 3.—The inverse relationship between the fluorescence intensity (—○—, —□—) and the fluorescence polarization (---●---, ---■---) after exposure to fluorescein diacetate.

FIG. 4.—The values of fluorescence intensity and fluorescence polarization of individual lymphocytes before (●) and after (○) stimulation by CaBP.

start of the measurement under the conditions for this study. SCM was therefore measured during that interval in this study.

The P values of individual lymphocytes from a patient with cancer are plotted against the fluorescence intensity before and after stimulation by CaBP as shown in Fig. 4. Similar results could not be obtained in PHA-stimulated lymphocytes because the clumps of agglutinated lymphocytes disturbed the measurement of fluorescence intensity. The distribution of activated lymphocytes was different from that of unstimulated lymphocytes in the experiment on CaBP stimulation. The fluorescence intensities and P values were measured every 2 min in identical unstimulated lymphocytes. The inverse relationship between the 2 components is presented in Fig. 3.

This phenomenon was observed repeatedly in unstimulated mouse spleens as well as cultured mouse lymphoma cells (L5178Y) and this result prompts us to make the following suggestion on the physicochemical mechanism of the SCM test. The P value of each cell is an average value determined by the distribution of the fluorescein in many intracellular sites. An increase in the amount of intracellular fluorescein will saturate some of the binding sites in the cytoplasm. Consequently, relatively more fluorescein will be dissolved in free water and this will decrease the fluorescence polarization. We think that increased average fluorescence intensity, with consequent lowering of polarization, may be one factor lowering the average P value of CaBP-treated cells compared to controls. However, this cannot be the only factor, since when CaBP-treated and control cells are compared at *equal* fluorescence intensities, there is still a lower average P value in the CaBP-treated population.

In another system—the Hitachi flow polarization meter—we have found that PHA-stimulated lymphocytes gave higher pulse height accompanied by lower P values than unstimulated lymphocytes. The relationships between fluorescent intensity and fluorescence polarization in stimulated lymphocytes should be further studied for the elucidation of the mechanism of the SCM test.

We are grateful to Drs L. and B. Cercek for their technical advice and to Mr K. Onogi and Mr N. Itoi for constructing the microscope. We wish to thank the surgeons of The Second Department of Surgery, Faculty of Medicine, University of Tokyo, for supplying the samples, and Mr T. Itoh and Miss S. Kakihara for their technical assistance. We are grateful to Miss K. Yoshioka for skilful typing of this manuscript.

### REFERENCES

CARNEGIE, P. P., CASPARY, E. A. & FIELD, E. J. (1973) Isolation of an "antigen" from malignant tumours. *Br. J. Cancer*, **28** (Suppl. 1), 219.

CERCEK, L. & CERCEK, B. (1976) Changes in the structuredness of cytoplasmic matrix (SCM) in human lymphocytes induced by PHA and cancer basic protein as measured in single cells. *Br. J. Cancer*, **33**, 539.

CERCEK, L., CERCEK, B. & FRANKLIN, C. I. V. (1974*b*) Biophysical differentiation between lymphocytes from healthy donors, patients with malignant diseases and other disorders. *Br. J. Cancer*, **29**, 345.

CERCEK, L., CERCEK, B. & GARRET, J. V. (1974*a*) Biophysical differentiation between normal human and chronic lymphocytic leukemia lymphocytes. In *Lymphocyte Recognition and Effector Mechanisms*. Ed. K. Lindahl-Kiessling & K. Osoba. New York: Academic Press. p. 553.

CERCEK, L. & CERCEK, B. (1977) Application of the phenomenon of changes in the structuredness of cytoplasmic matrix (SCM) in the diagnosis of malignant disorders: a review in *Perspectives in Cancer Research*. *Eur. J. Cancer*, **13**, 903.

HARRIS, R. & UKAEJIOFO, E. O. (1969) Rapid preparation of lymphocytes for tissue typing. *Lancet*, ii, 327.

PAUL, J. (1970) *Cell and Tissue Culture*. Edinburgh: E. & S. Livingstone. p. 91.

TAKAKU, F., YAMANAKA, T. & HASHIMOTO, Y. (1977) Usefulness of the SCM test in the diagnosis of gastric cancer. *Br. J. Cancer*, **36**, 810.


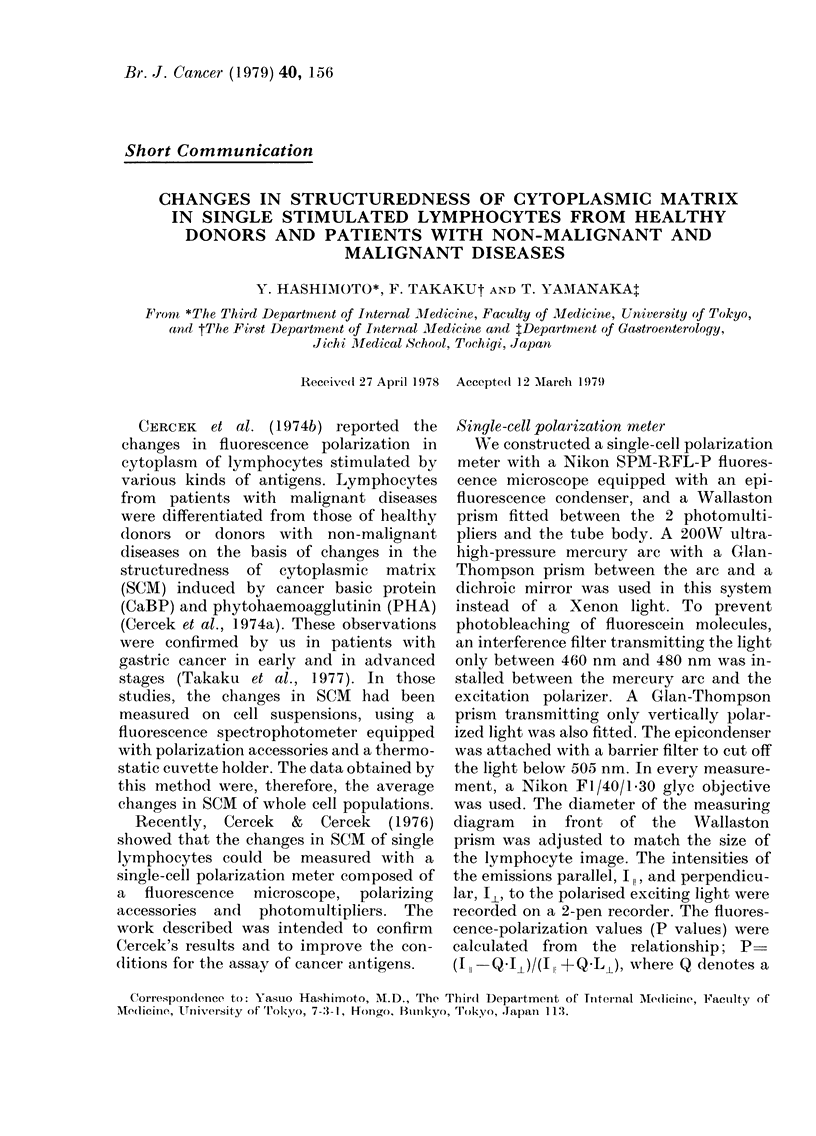

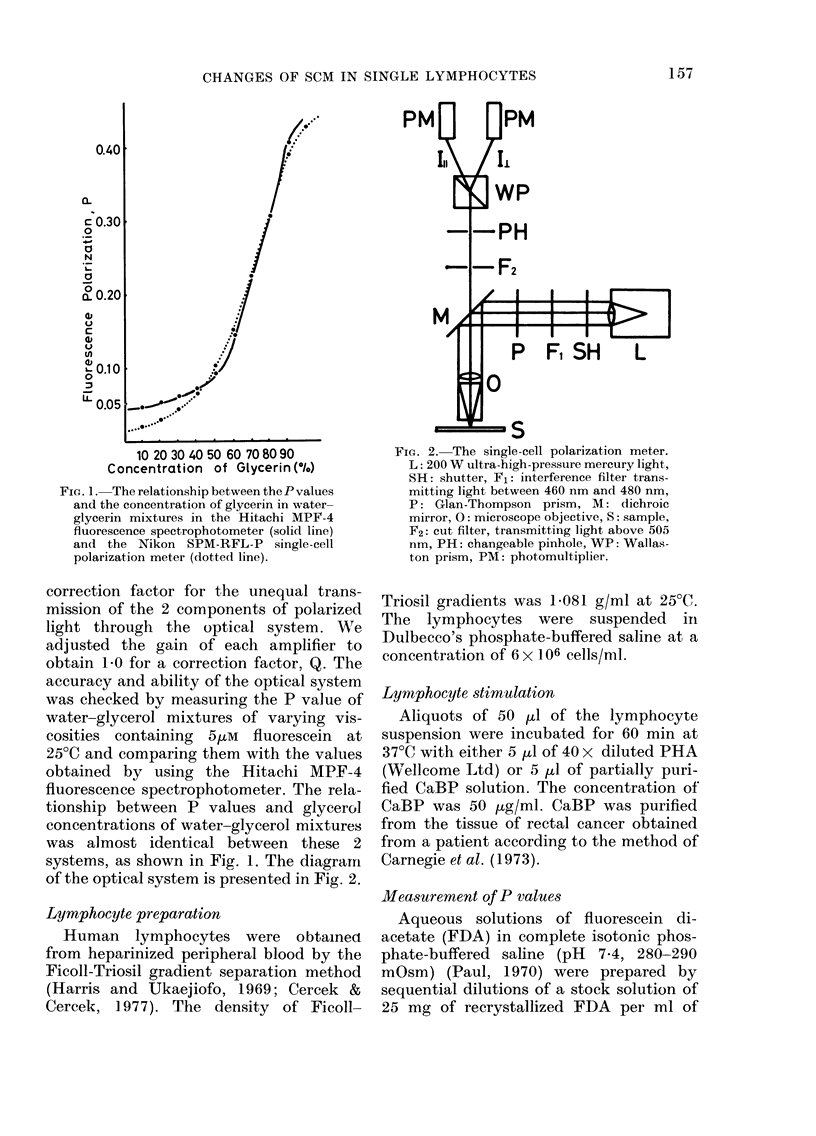

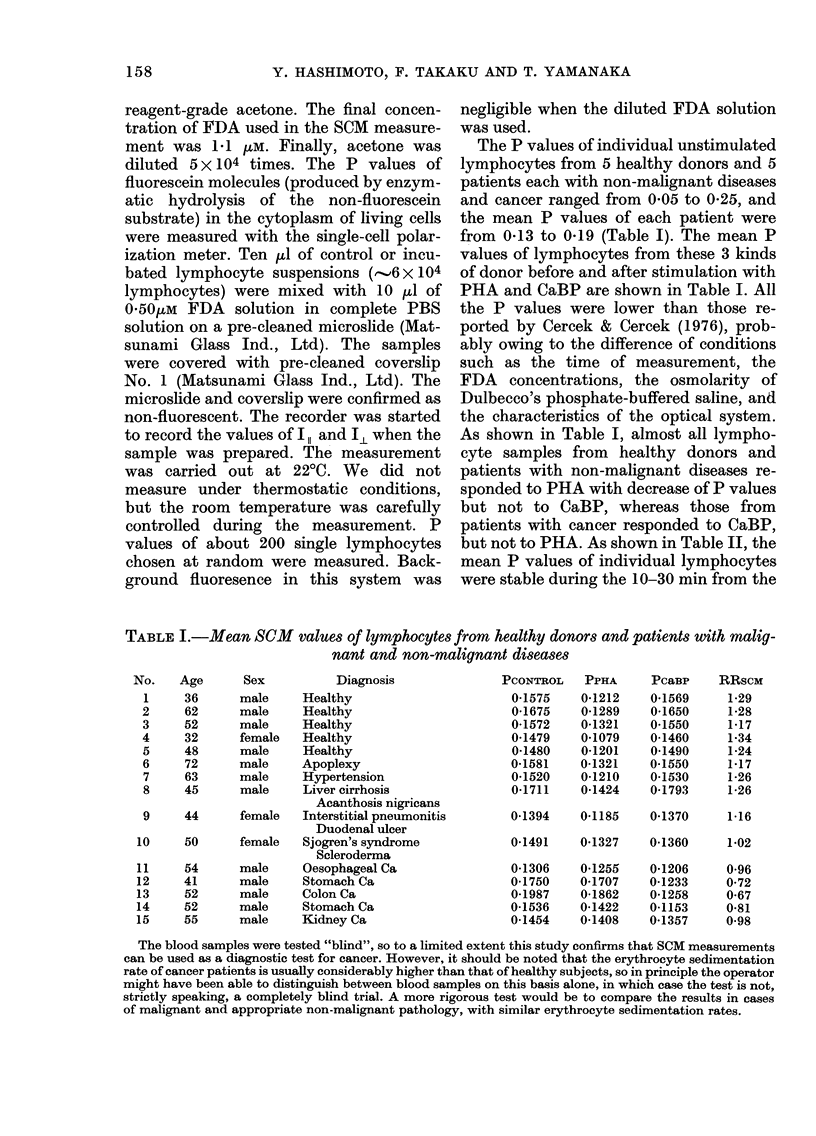

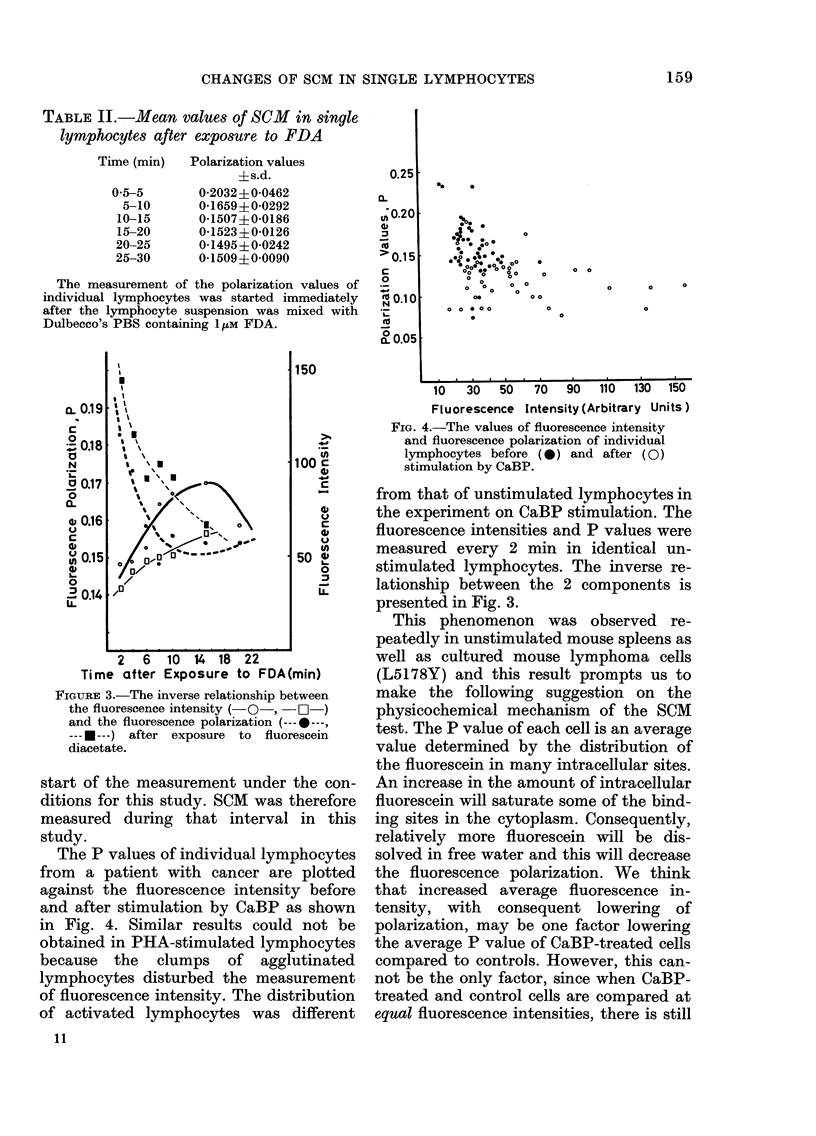

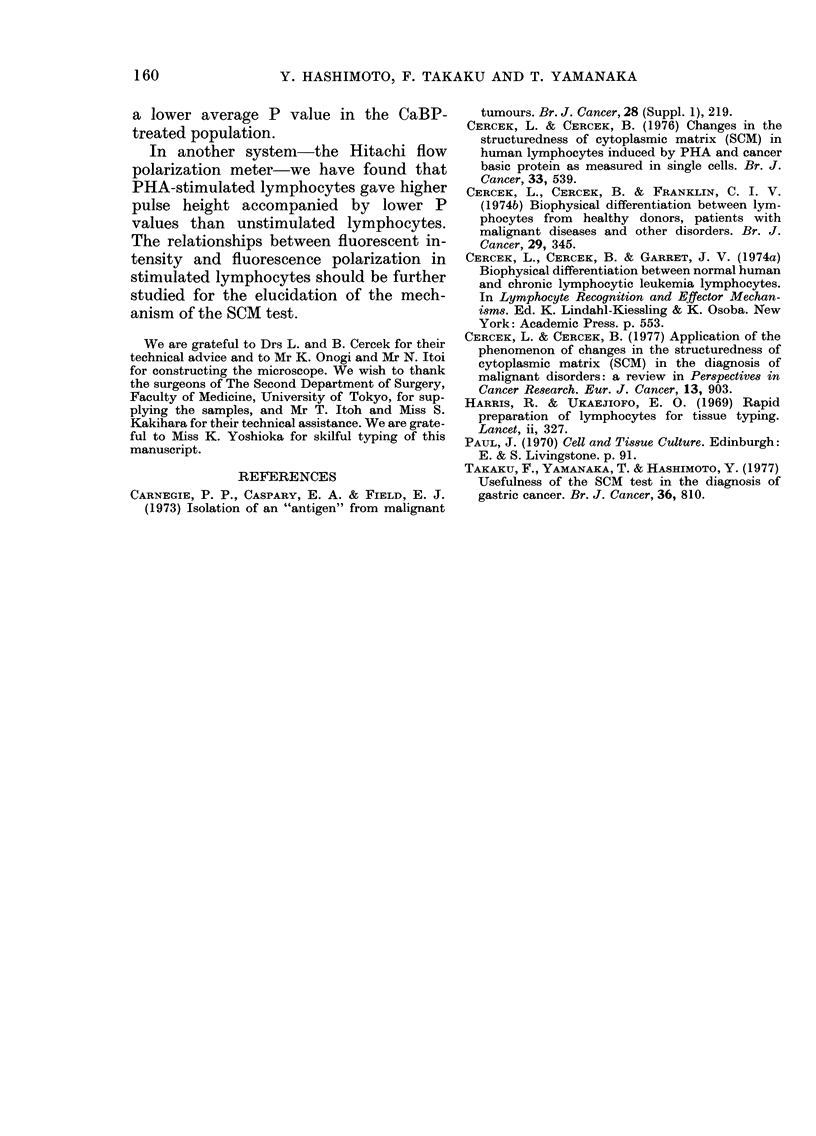


## References

[OCR_00636] Carnegie P. R., Caspary E. A., Field E. J. (1973). Isolation of an "antigen" from malignant tumours.. Br J Cancer Suppl.

[OCR_00664] Cercek L., Cercek B. (1977). Application of the phenomenon of changes in the structuredness of cytoplasmic matrix (SCM) in the diagnosis of malignant disorders: a review.. Eur J Cancer.

[OCR_00642] Cercek L., Cercek B. (1976). Changes in the structuredness of cytoplasmic matrix (SCM) in human lymphocytes induced by PHA and cancer basic protein as measured in single cells.. Br J Cancer.

[OCR_00649] Cercek L., Cercek B., Franklin C. I. (1974). Biophysical differentiation between lymphocytes from healthy donors, patients with malignant diseases and other disorders.. Br J Cancer.

[OCR_00671] Harris R., Ukaejiofo E. O. (1969). Rapid preparation of lymphocytes for tissue-typing.. Lancet.

[OCR_00680] Takaku F., Yamanaka T., Hashimoto Y. (1977). Usefulness of the SCM test in the diagnosis of gastric cancer.. Br J Cancer.

